# The Men Who Are Promoted

**Published:** 1884-11

**Authors:** 


### The Men Who are Promoted.

The *Manufacturers' Gazette*, in a recent editorial, made the following statements, regarding young men and their advancement, which others than the class to whom it is addressed will do well to heed :

" The young men who receive promotion are the men who do not drink on the sly. They are not the men who are always at the front whenever there is any strike, nor are they the men who watch for the clock to strike twelve, and leave their picks hanging in the air. They are not the men who growl if they are required to attend to some duty a few minutes after the whistle has sounded. They are the men usually who pay the closest attention to the details of their business, who act as if they were trying to work for their employer's interest instead of to beat him at every crook and turn. They are the men who give the closest attention to every practical detail, and who look continually to see whether they can do any better or not. This class of men are never out of a job. They are scarce. They never strike, they never loaf, and they do not ask for their pay two or three weeks before pay day."

